# Prioritizing mosquito-borne diseases during and after the COVID-19 pandemic

**DOI:** 10.5365/wpsar.2020.11.3.017

**Published:** 2021-05-07

**Authors:** Shahmshad Ahmed Khan, Cameron Ewart Webb, Nur Faeza Abu Kassim

**Affiliations:** a Department of Entomology, Faculty of Crop and Food Sciences, Pir Mehr Ali Shah (PMAS) Arid Agriculture University Rawalpindi, Shamsabad, Pakistan.; b Marie Bashir Institute for Infectious Diseases and Biosecurity, University of Sydney, Camperdown, Australia.; c 129 Medical Entomology Laboratory, School of Biological Sciences, Universiti Sains Malaysia, Minden, Penang, Malaysia.

The world is facing serious health and economic threats from the global coronavirus disease 2019 (COVID-19) pandemic, caused by severe acute respiratory syndrome coronavirus 2 (SARS-CoV-2). The burden of disease has been significant, with tens of millions of cases and more than 1.5 million deaths reported globally. (*1*) Since its emergence in Wuhan, China, in late 2019, COVID-19 has spread around the world, affecting almost all countries. COVID-19 is a highly contagious disease that is spread by direct contact and respiratory droplets, and patients can be infective while presymptomatic or asymptomatic. (*2*) To reduce opportunities for transmission, most developed countries have implemented lockdowns, causing significant social and economic disruption. Mosquito-borne diseases, such as malaria and dengue, are a substantial burden in many countries, especially those with developing economies. Malaria is the most significant mosquito-borne disease, with about 228 million cases reported in 2018 and 231 million in 2017, and 405 000 deaths in 2018 and 416 000 in 2017. (*3*) Dengue is the most commonly reported arboviral disease internationally, with Asia suffering a significant disease burden. (*4*) In countries facing endemic and epidemic malaria and dengue, disruption to government services (in health and non-health sectors) and to public health services could severely impact the ability to implement strategic responses to mosquito-borne diseases. As of 30 June 2020, all malaria-endemic countries in Asia had confirmed cases of COVID-19, and those with developing economies face a particularly serious threat to malaria control efforts. In these countries, local authorities responsible for malaria and dengue control must make strategic preparations for continuing with control measures, both during and after the COVID-19 pandemic.

Malaria and dengue control programmes in developing countries mainly focus on vector control by residual spraying of insecticides (other strategies include biological control of vectors and use of personal insect repellents and long-lasting insecticide-treated bed nets). (*5*) Between 2000 and 2015, malaria-endemic countries achieved remarkable success in malaria control, seeing about 60% reduction in malaria deaths and 37% reduction in cases. However, disrupting factors (e.g. war) can weaken malaria control programmes and result in a resurgent burden of malaria. (*6*)

Currently, there is uncertainty about the potential effects of the COVID-19 pandemic on existing malaria and dengue control programmes. For example, the dire global economic situation due to COVID-19 may reduce the ability of donor countries to continue their support of malaria and dengue control programmes in developing countries.

In recent years, donor countries have decreased their funding of malaria control programmes, prioritizing countries with higher disease burden; in addition, the resources available domestically for malaria and dengue control are limited. In many developing countries, malaria and dengue are major public health problems, with annual budgetary needs in the millions of dollars. However, control of these diseases is beneficial; for example, the 5-year growth of countries after malaria elimination is significantly more than in countries where malaria persists. (*7*) There is a precedent for emerging epidemics disrupting the response to existing public health threats. For example, the emergence of dengue in malaria-endemic countries can adversely affect malaria control programmes (e.g. the 2010 outbreak of dengue in Pakistan led to 702 000 more malaria cases in 2011). (*8*) It is already a challenge for countries to manage these two mosquito-borne diseases, with control often needing to be vector specific based on the distinct ecological requirements of the different mosquitoes. (*9*) Countries now face the challenge of focusing on dengue and malaria control during the COVID-19 pandemic. There is some uncertainty regarding how the COVID-19 pandemic will influence transmission rates of mosquito-borne pathogens. With the disruption to government services (e.g. through lockdowns or redeployment of government officials), control activities such as source reduction, community education and distribution of bed nets may cease or be significantly reduced. In residential and commercial buildings, efforts to reduce the risk of COVID-19 by creating more outdoor facilities and increasing circulation of indoor air may increase exposure to mosquitoes. Additionally, increased confinement at home during lockdowns, especially in metropolitan regions, may increase the risk of dengue virus transmission. If appropriate financial support is not maintained, the effectiveness of malaria and dengue control programmes will be compromised. Recent outbreaks of dengue have demonstrated the importance of adequately funding and implementing response strategies. In 2019, numbers of confirmed dengue cases increased compared with previous years in many countries in Asia and the Pacific, including Malaysia, the Philippines and Viet Nam. (*10*)

There is a need to distribute resources to simultaneously control dengue, malaria and COVID-19 in malaria-endemic countries. Given the possibility of reduced funding from donor countries, governments should consider earmarking funds for the support of malaria and dengue control programmes, both during and after the pandemic. The COVID-19 pandemic is likely to lead to strategic changes to public health policies in many countries, but prioritizing control of mosquito-borne diseases will remain critical. Many aspects of integrated mosquito control can be incorporated into existing and future public health strategies. These include community and household efforts to increase the use of sanitary water storage practices in homes, use of personal protection measures (e.g. bed nets and repellents) and protection of vulnerable populations (e.g. pregnant women, young children and older people). There has been significant international collaboration to develop responses to COVID-19. If the increased awareness of the importance of public health can lead to a greater focus on developing responses to mosquito-borne disease, there may be a positive outcome from the current situation. Although there may be competing public health priorities, especially for COVID-19, authorities must maintain the programmes designed to reduce the burden of malaria and dengue.
